# High-Frequency Oscillations on Interictal Epileptiform Discharges in Routinely Acquired Scalp EEG: Can It Be Used as a Prognostic Marker?

**DOI:** 10.3389/fnhum.2021.709836

**Published:** 2021-07-30

**Authors:** Hanan El Shakankiry, Susan T. Arnold

**Affiliations:** UT Southwestern Medical School, Children’s Health, Dallas, TX, United States

**Keywords:** high-frequency oscillations, ripples, scalp EEG, prognostic marker, epilepsy

## Abstract

**Introduction:**

Despite all the efforts for optimizing epilepsy management in children over the past decades, there is no clear consensus regarding whether to treat or not to treat epileptiform discharges (EDs) after a first unprovoked seizure or the optimal duration of therapy with anti-seizure medication (ASM). It is therefore highly needed to find markers on scalp electroencephalogram (EEG) that can help identify pathological EEG discharges that require treatment.

**Aim of the study:**

This retrospective study aimed to identify whether the coexistence of ripples/high-frequency oscillations (HFOs) with interictal EDs (IEDs) in routinely acquired scalp EEG is associated with a higher risk of seizure recurrence and could be used as a prognostic marker.

**Methods:**

100 children presenting with new onset seizure to Children’s Medical Center- Dallas during 2015–2016, who were not on ASM and had focal EDs on an awake and sleep EEG recorded with sample frequency of 500 HZ, were randomly identified by database review. EEGs were analyzed blinded to the data of the patients. HFOs were visually identified using review parameters including expanded time base and adjusted filter settings.

**Results:**

The average age of patients was 6.3 years (±4.35 SD). HFOs were visually identified in 19% of the studied patients with an inter-rater reliability of 99% for HFO negative discharges and 78% agreement for identification of HFOs. HFOs were identified more often in the younger age group; however, they were identified in 11% of patients >5 years old. They were more frequently associated with spikes than with sharp waves and more often with higher amplitude EDs. Patients with HFOs were more likely to have a recurrence of seizures in the year after the first seizure (*P* < 0.05) and to continue to have seizures after 2 years (*P* < 0.0001). There was no statistically significant difference between the two groups with regards to continuing ASM after 2 years.

**Conclusion:**

Including analysis for HFOs in routine EEG interpretation may increase the yield of the study and help guide the decision to either start or discontinue ASM. In the future, this may also help to identify pathological discharges with deleterious effects on the growing brain and set a new target for the management of epilepsy.

## Introduction

Despite all the efforts for optimizing epilepsy management in children over the past decades, controversy still exists in many areas in this field; there is no clear consensus regarding whether or not to treat epileptiform discharges (EDs), which are seen on electroencephalogram (EEG), or the duration of therapy.

For patients presenting with their first seizure, it is well known that the underlying etiology and whether the EEG is normal or abnormal are factors consistently related to the risk of recurrence; however, randomized controlled trials have demonstrated that compared to no or delayed treatment, antiepileptic drugs reduce the risk of a second seizure but do not alter longer-term seizure outcomes (Hirtz et al., 2003; Marson, 2008), and there is no existing EEG prognostic marker for intractability. In addition, when epilepsy is in remission, the optimal timing of anti-seizure medication (ASM) discontinuation is still unknown (Strozzi et al., 2015).

It is also worth mentioning that EDs have been observed in 0.6 and 7.0% of children without epileptic seizures and relatives of patients with epilepsy (Bali et al., 2007; Borusiak et al., 2010). The high incidence of abnormal patterns in the EEGs of normal people has detracted from the perceived value of EEG, perhaps unfairly. It had been postulated that subclinical discharges may be “ictal” to higher cortical functions and that many developmental or acquired defects of language or behavior (e.g., Autism) are a consequence of apparently subclinical spikes that may impair the neural processes and possibly the local plastic changes associated with learning and cognition (Guerrini et al., 2002; Laporte et al., 2002; Pressler et al., 2005; Levisohn, 2007; El Shakankiry, 2010; Bermeo-Ovalle, 2018); however, on the contrary, there are epileptic syndromes with frequent EDs in EEG but with no or minimal seizure recurrence risk and no or minimal effect on cognition. Defining what should be considered as a pathological discharge on scalp EEG is, therefore, highly needed. Clinicians rely on the location and morphology of EDs to determine the risk of seizure recurrence but identifying additional characteristics as predictive markers for prognosis could improve the yield of EEG as a tool, revolutionizing the concept of epileptogenicity and the management of epilepsy.

Over the past three decades, the rise of broadband digital systems has enabled the monitoring of electroencephalographic signals beyond the classical Berger frequency band (0.3–70 Hz), extending the recordings to frequencies as high as 500 Hz and beyond. High-frequency oscillations (HFOs) include all physiologic and pathologic oscillatory activities within the frequency band from the 80 to 500 Hz range that stands out from the baseline and persists for at least four oscillation cycles. They are classified into ripples (80–250 Hz) and fast ripples (FR, >250 Hz) (Zijlmans et al., 2017).

High-frequency oscillations were initially identified in recordings with depth and grid electrodes. In animal studies, they were linked to spontaneous seizures (Bragin et al., 2004). More recent studies have established that HFOs are more specific in identifying the seizure onset zone than traditional spikes and are more tightly linked to seizures than spikes alone (Jacobs et al., 2008).

Studies using depth electrodes, grids, or intraoperative corticography showed that resection of HFO-generating tissue and particularly disconnecting networks generating HFOs correlate with a favorable post-surgical seizure outcome (Akiyama et al., 2011; Usui et al., 2011; van Klink et al., 2014; van ’t Klooster et al., 2017).

It was initially believed that the skull filters away higher frequencies, and it was not expected to find epileptic HFOs with scalp EEG; however, this proved to be incorrect, as the skull does not have low-pass filter capacities. It diminishes signal amplitude due to the greater distance between generators and sensors, but as long as the signal amplitude is greater than noise, the high-frequency signal can be distinguished on a scalp EEG recording. A comparative study with simultaneous scalp and subdural electrodes showed that HFOs co-occurred in both simultaneously (Zelmann et al., 2014; Zijlmans et al., 2017).

In the past decade, HFOs have been identified in the scalp EEG; however, investigation and clinical application of HFOs as a biomarker for epilepsy have been limited. Recent studies demonstrated that HFOs co-existing with EDs are easier to detect than HFOs alone and have greater pathological significance (Kramer et al., 2019). Physiologic ripples have not been reported in scalp recordings which may implicate that they may be attenuated before they reach the scalp (Von Ellenrieder et al., 2014).

This retrospective study aims to identify whether the electrographic finding of HFOs in coexistence with interictal spikes on routinely acquired scalp EEG is associated with a higher risk of seizure recurrence than interictal spikes alone and could therefore be used as a prognostic marker on a scalp EEG recording.

## Materials and Methods

### A-Subjects

Children presenting with new onset seizure to Children’s Medical Center Dallas, during 2015–2016, who were not on ASM and had focal ED on an awake and sleep EEG recorded with a sample frequency of 500 HZ, were identified by review of an electronic database. 100 children were randomly selected and enrolled in the study.

### B-Methods

Charts of the included patients were reviewed with regard to the age at seizure onset, type/s of seizures, the start of ASM, brain imaging result, and diagnosed epilepsy syndrome if any.

Charts of the patient who continued to follow-up in the clinic for 2 years were reviewed with regards to the presence or absence of seizures during the 1st year and after 2 years of follow-up, identified cause for seizure recurrence and ASM after 2 years of follow-up.

### EEG Acquisition and Analysis

**FIGURE 1:**
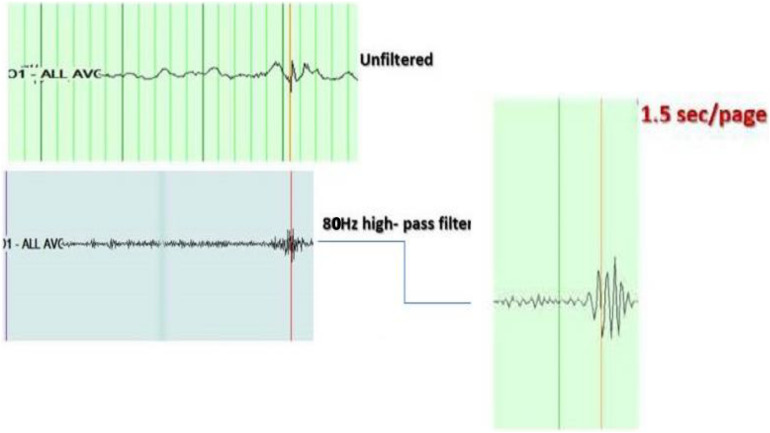
Example of high-frequency oscillations (HFOs) on top of the spike. Top right: shows the spike at O1 in unfiltered EEG, viewed with a time base of 15 mm/s, a high-pass filter of 0.5 Hz, a low-pass filter of 70 Hz, notch filter of 60 Hz, and a sensitivity of 15 uV/mm. Middle right: shows the same spike with a time base of 60 mm/s, a high-pass filter of 80 Hz, and a sensitivity of 1 uV/mm. Left: shows the HFOs viewed with an expansion of the EEG trace to 1.5 s/page.

**FIGURE 2:**
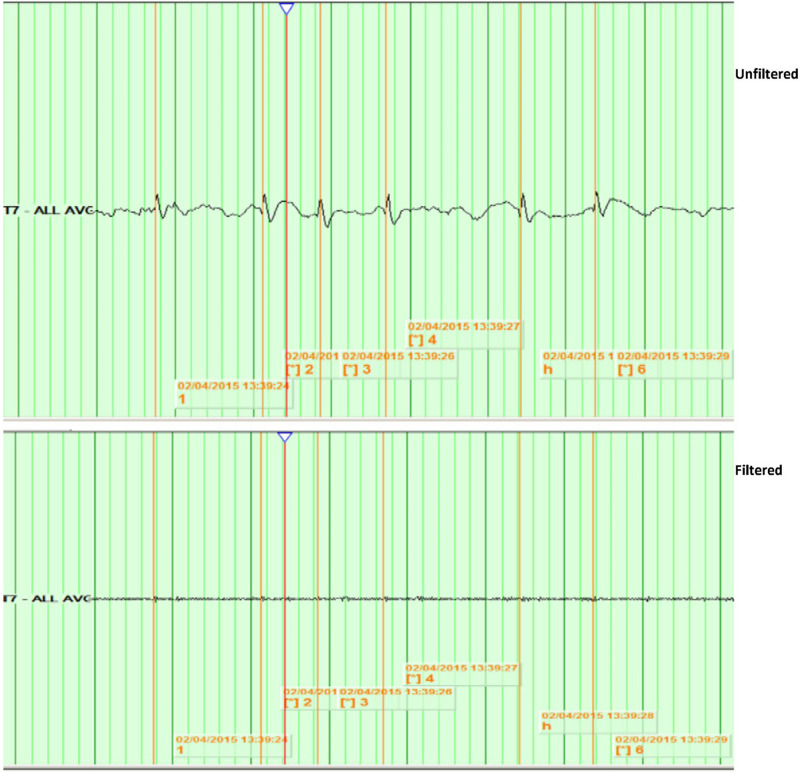
Example of no identified HFOs. Top: shows spikes at T7 in unfiltered EEG, viewed with a time base of 15 mm/s, a high-pass filter of 0.5 Hz, a low-pass filter of 70 Hz, notch filter of 60 Hz, and a sensitivity of 15 uV/mm. Bottom: shows the same spikes with a time base of 60 mm/s, a high-pass filter of 80 Hz, and a sensitivity of 1 uV/mm.

– Awake and sleep EEG was recorded for each patient with 21 channels and a sampling frequency of 500 Hz using XLTEK Natus EEG system, CA, United States, version 988. The standard international 10–20 electrode system was used. EEGs were analyzed blinded to data of patients.
– EEGs were reviewed in bipolar and referential montages, using as a reference the average of the 21 electrodes, unfiltered, with a time base of 15 mm/s, a high-pass filter of 0.5 Hz, a low-pass filter of 70 Hz, notch filter of 60 Hz, and a sensitivity of 15 uV/mm.
– EEGs were reviewed for the presence of background slowing and asymmetry. The EEGs were classified as either with or without background abnormality.
– Epileptiform discharges were selected from portions of the EEG which were free from any muscle artifact, often during drowsiness or sleep. We analyzed every ED in the artifact-free portions of the EEGs studied. Because in some EEGs we could not identify more than three EDs in the artifact-free portions, we decided to choose three representative EDs at least 1 s apart for each patient from an artifact-free portion of the recording to analyze.
– Channels with the highest amplitude of EDs were identified; the duration, amplitude, and topography of each discharge were recorded.
– High-frequency oscillations superimposed on EDs were identified visually by the first investigator through temporal expansion of the EEG traces with a time base of 60 mm/s, a high-pass filter of 80 Hz, and a sensitivity of 1 uV/mm, and then viewed at 1.5 s/page. HFOs were defined as oscillatory events with at least four cycles above 80 Hz, which are visible above the background signal in the filtered data (examples in Figures 1, 2).

– EEGs were then reviewed by the second investigator to establish inter-observer reliability.

### Statistical Analysis

Data were analyzed for the distribution and characteristics of the EDs associated with HFOs. Patients who had coexistence of HFOs with interictal EDs (IEDs) on scalp EEG were compared with those with no HFOs with regards to brain imaging results, the incidence of background abnormality in EEG, the topography of the EDs, initiation and duration of ASM, seizure recurrence after 1 year and after 2 years of follow-up. Data were analyzed for statistical significance using unpaired *t*-test comparison, a *p*-value of <0.05 was considered significant.

This study was approved by the Institutional Review Board (IRB) of The University of Texas Southwestern Medical Center, Dallas, TX, United States.

## Results

**FIGURE 3:**
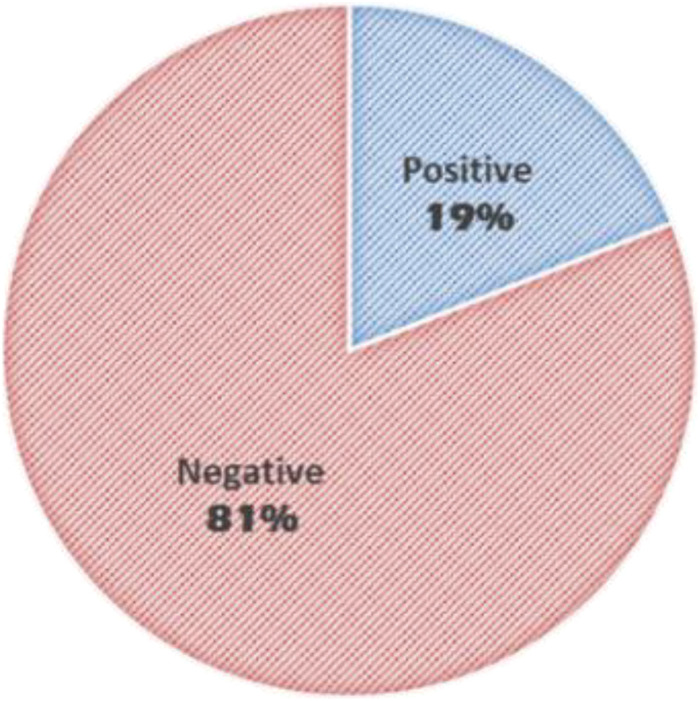
Prevalence of HFOs among studied patients.

The average age of patients was 6.3 years (±4.35 SD). HFOs were visually identified in 19% of the studied patients (Figure 3). Inter-rater reliability for identification of HFO negative discharges was 99% and for identification of HFOs agreement was 78%.

**FIGURE 4:**
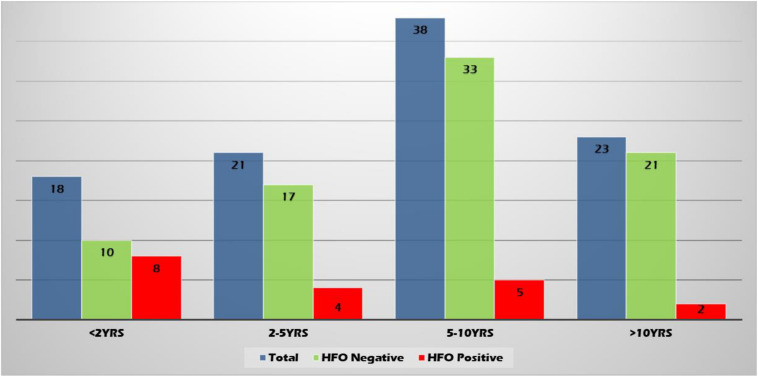
Age group representation.

High-frequency oscillations were found more often in the younger age group (22.2, 9.5, 6.58, and 4.35% of patients in age groups <2 years, 2–5 years, 5–10 years, and >10 years, respectively) (Figure 4).

**FIGURE 5:**
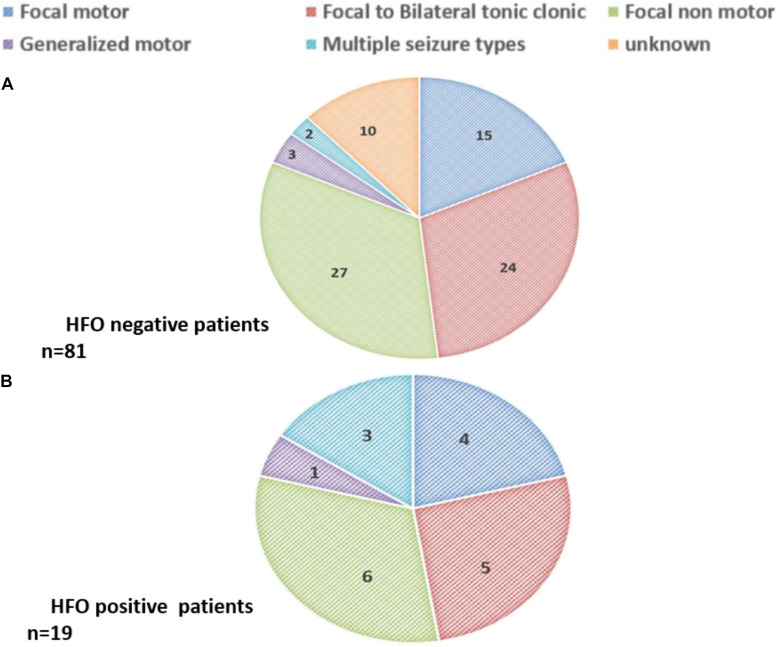
Seizure types among **(A)** HFO negative patients (*n* = 81) and **(B)** HFO positive patients (*n* = 19).

Types of seizures in each group are shown in Figure 5. Most patients had focal seizures; focal non-motor seizures were the most common type seen in both groups.

**FIGURE 6:**
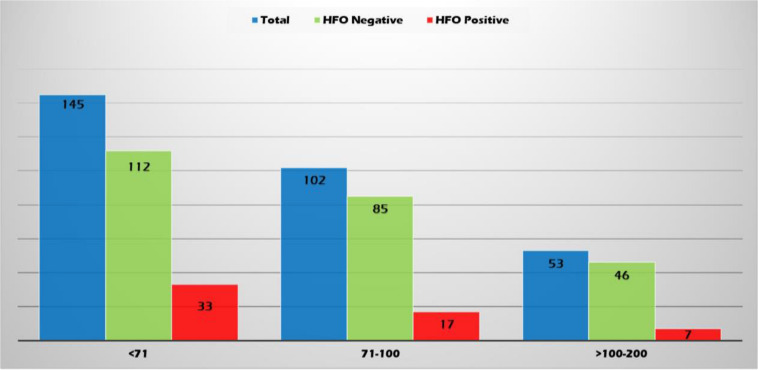
Relationship between epileptiform discharge (ED) duration (ms) and presence of HFOs.

**FIGURE 7:**
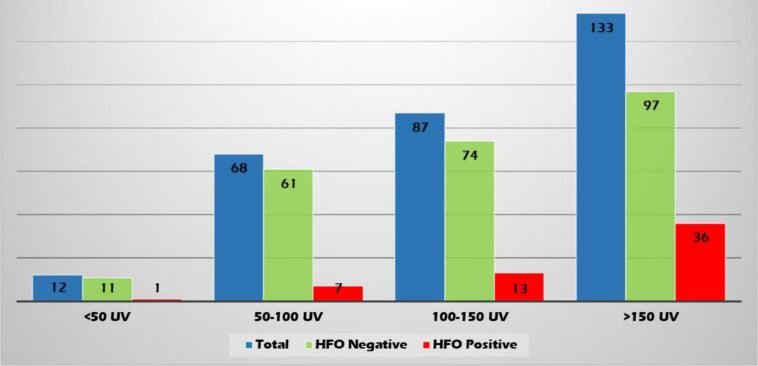
Relationship between ED amplitude and presence of HFOs.

**FIGURE 8:**
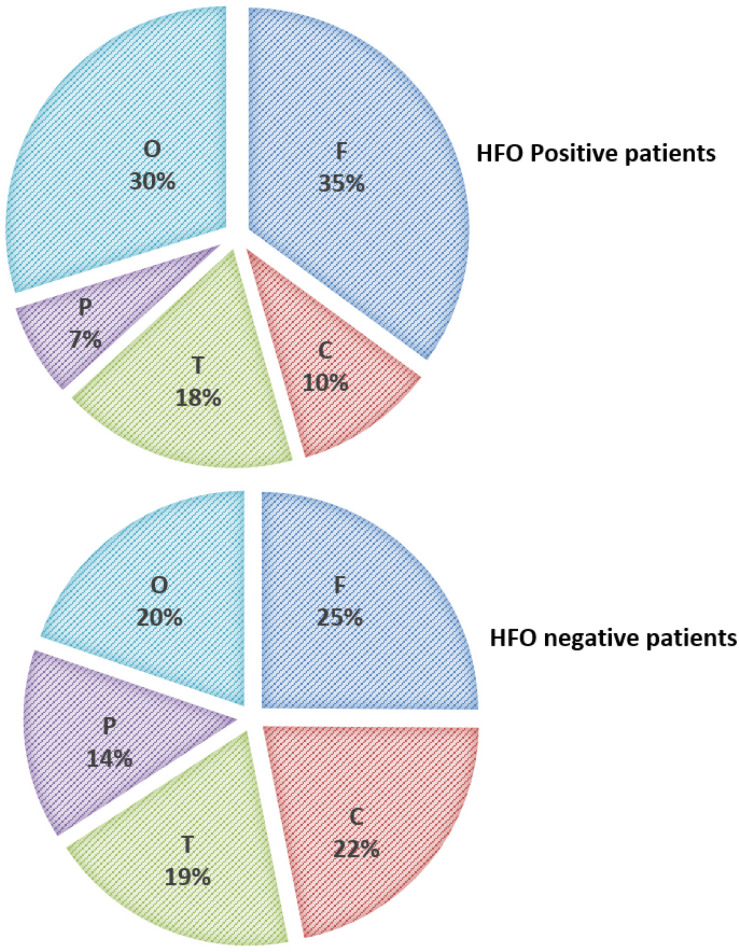
Topographic distribution of ED in HFO positive and negative patients (F, Frontal; C, Central; T, Temporal; P, Parietal; and O, Occipital).

High-frequency oscillations were identified with both spikes or sharp waves; however, they were more frequently associated with spikes (11.4, 8.3, and 6.6% of the EDs with a duration of <71, 71–100, and >100–200 ms, respectively) (Figure 6). They were identified more often with higher amplitude spikes/sharp waves (7.4% for EDs with amplitude 100–150 uV and 13.5% for those >150 uV compared to 4.1% for EDs < 50 uV and 5.1% for EDs 50–100 uV) (Figure 7). There was no significant difference in the topographic distribution of the EDs among HFO positive and negative patients (Figure 8).

There was no statistically significant difference between the two groups with regards to the presence of background abnormalities (7/19 of HFO positive patients and 28/81 of HFO negative patients). There was also no difference in the presence of generalized ED in addition to the focal discharges (3/19 of HFO positive patients and 8/81 of HFO negative patients) and the presence of multifocal EDs vs. a single electrographic focus (10/19 of HFO positive patients and 38/81 of HFO negative patients).

Two of the HFO positive patients (10.5%) and fifteen of the HFO negative patients (18.5%) were diagnosed with benign rolandic epilepsy with centrotemporal spikes (BECTs). Structural causes and epileptic encephalopathies were more common among the younger age group.

Brain imaging was done in 71/81 (87.7%) of the HFO negative patients (2 had brain CT and 69 had Brain MRI done). All 19 HFO positive patients had Brain MRI performed. Imaging was abnormal in 36.6% of the HFO negative patients (26/71 patients) and 52.6% of the HFO positive patients (10/19 patients). The difference between the two groups was not statistically significant (*P* > 0.05).

15/19 HFO positive and 69/81 HFO negative patients continued to follow-up for 1 year and 15/19 HFO positive and 59/81 HFO negative patients continued to follow-up for 2 years. Patients with HFOs were more likely to have recurrence of seizures during the 1st year after diagnosis, 93.3 vs. 39.1% for HFO negative patients (*P* < 0.05) (Table 1) and to continue to have seizures after 2 years, 86.7 vs. 15.3% (*P* < 0.0001) (Table 2). There was no statistically significant difference between the two groups with regards to continuing on ASM after 2 years (Table 3).

**TABLE 1:** Seizure recurrence during the 1st year of follow-up.

**Seizure recurrence during 1st year**	**HFO positive (*n* = 15)**	**HFO negative (*n* = 69)**	
No	1 (6.7%)	42 (60.9%)	
Yes			
**Unprovoked**			
Single	1	10	
Multiple	10	9	
**Provoked**	3	8	
Total	14 (93.3%)	27 (39.1%)	***P*-value < 0.05**

*Bold represents that the values are statistically significant.*

**TABLE 2:** Seizure recurrence after 2 years of follow-up.

**Seizure recurrence after 2nd year**	**HFO positive (*n* = 15)**	**HFO negative (*n* = 59)**	
No	2 (13.3%)	50 (84.7%)	
Yes			
Unprovoked	13	5	
Provoked	0	4	
Total	13 (86.7%)	9 (15.3%)	***P*-value < 0.0001**

*Bold represents that the values are statistically significant.*

**TABLE 3:** Number of patients on anti-seizure medication (ASM) after 2 years of follow up.

**ASM after 2nd year**	**HFO positive (*n* = 15)**	**HFO negative (*n* = 59)**	
No ASM	3 (20%)	25 (42.4%)	
On ASM	12 (80%)	34 (57.6%)	***P*-value > 0.05**

*Bold represents that the values are statistically significant.*

## Discussion

Scalp-recorded HFOs were reported ictally, at the onset of epileptic spasms (Kobayashi et al., 2004) and in absence seizures (Chaitanya et al., 2015), and interictally in idiopathic partial epilepsy (Kobayashi et al., 2011), epilepsy with electrical status epilepticus in sleep (ESES) (Kobayashi et al., 2010), and atypical benign partial epilepsy (ABPE) (Qian et al., 2016); however, in these studies, EEGs were either recorded with high sampling rates, utilized time-frequency power spectral analysis with Gabor transform of the signal, and/or used semiautomated or fully automated analysis. The purpose of this study was to demonstrate that a simple visual method, with no additional software, could be used to identify HFOs during routine EEG interpretation in clinical practice to help identify patients with a higher risk of seizure recurrence. The EEGs studied were routinely acquired using the standard 21 channel system. While using more channels and high-density recordings would be ideal, these methods are not used routinely and are not available in all centers. We were able to visually identify HFOs in 19% of the studied routinely acquired scalp EEGs recorded with a sample frequency of 500 HZ, with high inter-rater reliability.

It is reported that whenever filters are applied there is a chance that the spike can also degrade into an irregular oscillatory pattern (Bénar et al., 2010); therefore, in this study, only those oscillations which showed a regular ripple with at least four cycles with amplitude being distinctly different from the background were considered as HFOs; this is in accordance with other previous studies (Zelmann et al., 2009; Zijlmans et al., 2012).

High-frequency oscillations were identified more often in the younger age group, yet, they were identified in 11% of patients >5 years old. Upon chart review, patients below 5 years of age were more commonly diagnosed with epileptic encephalopathy or structural cause whereas older patients were more commonly diagnosed with idiopathic epilepsy syndrome (BECT and Panayiotopoulos syndrome). Brain imaging was more likely to be abnormal in patients who were HFO positive compared to those who were HFO negative (52.6 and 36.6%, respectively); however, this difference was not statistically significant. Seventeen of our studied patients were diagnosed with BECT; two were HFO positive. It is well known that in BECT, the EDs often persist despite clinical seizure remission (Panayiotopoulos, 2007) further supporting the concept that additional factors determine ictogenesis.

On visual scanning of the unfiltered EEGs, we observed that neither the spike load nor the activation of the EDs during sleep could predict the presence of HFOs. Paroxysmal EDs augmented during sleep were previously reported in children with no clinical seizures (El Shakankiry, 2010) raising the question of how to identify active or pathological spikes that need to be treated. In this study, there was also no significant difference between HFO positive and negative patients with regards to the topographic distribution of the EDs or EEG background abnormality, this highlights that in clinical practice, one cannot predict from the appearance of interictal discharges which may have superimposed HFOs. It also implies that HFOs, when present, can be visually identified irrespective of the topographic distribution of the EDs.

High-frequency oscillations were identified with both spikes or sharp waves; however, they were more frequently associated with spikes and more often with higher amplitude EDs. Spikes and sharp waves are two types of IEDs; irrespective of their morphology both are assigned equal significance in the evaluation of a patient with epilepsy (Jaseja and Jaseja, 2012). In a study by van Klink et al. (2016) investigating the spatiotemporal relation between spikes and ripples and the difference between spikes that do and do not co-occur with ripples, spikes with ripples were reported to be shorter and had higher amplitude and higher slope than spikes without ripples.

The co-existence of HFOs with spikes or sharp waves was correlated with seizure recurrence. Patients with HFOs were more likely to have a recurrence of seizures during the 1st year after diagnosis and to continue to have seizures after 2 years. This agrees with previous studies reporting that HFOs were related to the epileptic disease activity (Zijlmans et al., 2009; Kerber et al., 2013; Kobayashi et al., 2015; Qian et al., 2016), respond to therapy more than the spikes (Qian et al., 2016), and could be a valuable predictor of the course of the disease (van Klink et al., 2016). HFOs and spikes were suggested to be two independent entities. From the perspective of cellular mechanisms, spikes reflect excitatory postsynaptic potentials (De Curtis and Avanzini, 2001), while HFOs likely reflect synchronized co-firing of small clusters of principal cells (Bragin et al., 2002, 2011). Recently, strategies aimed at improving the ability of principal neurons to maintain a *trans-*membrane chloride gradient in the face of excessive network activity were suggested to prevent interneurons from contributing to seizure perpetuation (Magloire et al., 2019; Raimondo and Dulla, 2019). Shifting target in epilepsy management to inhibition of principal cells cofiring is therefore worth studying, particularly that despite the availability of many new ASMs with differing mechanisms of action, the overall outcomes in newly diagnosed epilepsy have not improved (Chen et al., 2018).

There was no statistically significant difference between the two studied groups with regards to the initiation of ASM at presentation or continuing ASMs after 2 years; 57.6% of HFO negative patients were still on ASM after 2 years even though 84.7% of them were seizure-free. Hixson (2010) reported that after initiation of an ASM and achieving a sustained period of seizure freedom, the bias toward continuing therapy indefinitely can be substantial, and the required duration of antiepileptic therapy is much less concrete and remains controversial (Strozzi et al., 2015). This highlights the importance of applying HFO analysis as a marker of disease activity to guide the decision about discontinuing ASM to avoid undue prolonged duration of therapy without increasing the risk of seizure recurrence after discontinuing ASM.

## Conclusion

In this retrospective study, we found that patients with HFOs were more likely to have recurrence of seizures during the 1st year after diagnosis, 93.3 vs. 39.1% for HFO negative patients (*P* < 0.05) and to continue to have seizures after 2 years, 86.7 vs. 15.3% (*P* < 0.0001). Despite that, we found out that there was no statistically significant difference between the two groups with regards to the initiation of ASM at presentation or continuing ASMs after 2 years. The fact that there was no difference in the use of ASM between the two groups emphasizes the need for a prognostic marker to guide the clinical decision regarding starting or discontinuing ASM.

The international league against epilepsy defines epilepsy as two or more unprovoked seizures or a single unprovoked seizure if the recurrence risk is similar to the general risk after two unprovoked seizures (Fisher et al., 2014). The findings of this study suggest that the presence of HFOs superimposed on EDs is a significant risk factor for seizure recurrence. We, therefore, propose that the presence of HFOs in interictal discharges will be a helpful marker for clinicians to assess the recurrence risk. If the EEGs were analyzed for the presence or absence of HFOs prospectively, the two groups would have been treated differently; the HFO positive patients would have been on ASM, while many HFOs negative patients might have not been started on ASM or might have had the ASM discontinued earlier.

A limitation of this study arises from its retrospective design, and while the sample size of 100 children appeared adequate, the group with HFOs and 2 years follow-up was relatively small. Although a larger study would be ideal, we believe that the strong statistical significance in seizure recurrence rates between the groups shows that including analysis for HFOs in routine EEG interpretation may increase the yield of the EEG and help guide the decision to either start or discontinue ASM.

Further prospective studies applying this observation are needed to decide whether to start ASM after a new onset seizure or discontinue ASM after a period of seizure freedom and to identify pathological discharges with deleterious effects on the growing brain. This may also help set a new target for the management of refractory epilepsy.

## Data Availability Statement

The raw data supporting the conclusions of this article will be made available by the authors, without undue reservation.

## Ethics Statement

The studies involving human participants were reviewed and approved. The study was approved by the Institutional Review Board (IRB) The University of Texas Southwestern Medical Center, Dallas, TX, United States. Written informed consent for participation was not required for this study in accordance with the national legislation and the institutional requirements.

## Author Contributions

HE: research proposal, data collection, and preparation of the manuscript. SA: principal investigator, provided oversight, review, and mentorship. Both authors contributed to the article and approved the submitted version.

## Conflict of Interest

The authors declare that the research was conducted in the absence of any commercial or financial relationships that could be construed as a potential conflict of interest.

## Publisher’s Note

All claims expressed in this article are solely those of the authors and do not necessarily represent those of their affiliated organizations, or those of the publisher, the editors and the reviewers. Any product that may be evaluated in this article, or claim that may be made by its manufacturer, is not guaranteed or endorsed by the publisher.
